# Hodological patterning as an organizing principle in vertebrate motor circuitry

**DOI:** 10.3389/fnana.2024.1510944

**Published:** 2025-01-08

**Authors:** Joel C. Glover

**Affiliations:** Department of Molecular Medicine, Institute of Basic Medical Sciences, University of Oslo, Oslo, Norway

**Keywords:** motoneuron, preganglionic sympathetic neuron, spinal interneuron, reticulospinal, vestibulospinal, vestibulo-ocular, corticospinal

## Abstract

Hodological patterning refers to developmental mechanisms that link the location of neurons in the brain or spinal cord to specific axonal trajectories that direct connectivity to synaptic targets either within the central nervous system or in the periphery. In vertebrate motor circuits, hodological patterning has been demonstrated at different levels, from the final motor output of somatic and preganglionic autonomic neurons targeting peripheral motoneurons and ganglion cells, to premotor inputs from spinal and brainstem neuron populations targeting the somatic motoneurons and preganglionic autonomic neurons, to cortical neurons that delegate movement commands to the brainstem and spinal neurons. In many cases molecular profiling reveals potential underlying mechanisms whereby selective gene expression creates the link between location and axon trajectory. At the cortical level, somatotopic organization suggests a potential underlying hodological patterning, but this has not been proven. This review describes examples of hodological patterning in motor circuits and covers current knowledge about how this patterning arises.

## Introduction

Motor circuits provide targeted activation of musculature to generate appropriate movements in time and space. How activity in premotor neuron populations is channeled to motoneurons remains poorly understood. During vertebrate evolution, an increasing elaboration of body musculature, particularly with the advent of limbs, has been accompanied by an increase in the complexity of premotor connectivity. This enables a broad repertoire of preparatory and compensatory movements that stabilize principal movements within the large parameter space embodied by an interconnected system of moving parts. Within this “connectivity envelope” there must be central elements that define the principal movements, by linking specific populations of premotor neurons to specific sets of motoneurons. Indeed, in some reflexes, such as the inhibitory arm of the Ia afferent pathway in the spinal cord, and the vestibulo-ocular reflex pathway in the brain stem, central pathways with such specificity have been characterized. In a number of cases, higher order motor circuitry also exhibits inherently specific connectivity, suggesting a conserved wiring pattern that lies at the heart of context-appropriate function.

There are likely to be core organizing principles that dictate these central elements of connectivity based on developmental mechanisms governing neuron migration, settling, targeting and recognition. In this review, I focus on a feature of developmental patterning that likely plays a key role, and which is evident at several levels of premotor circuitry: hodological patterning. Hodological patterning involves a link between the regional expression of specific genes by differentiating neurons on the one hand, and the developing trajectories and termination patterns of their axons on the other. In this sense, hodological patterning provides a developmental record of how neuron identity at the molecular level is mapped, in broad strokes, to synaptic connections with targets.

## Hodological patterning of somatic motoneurons

**Figure 1:**
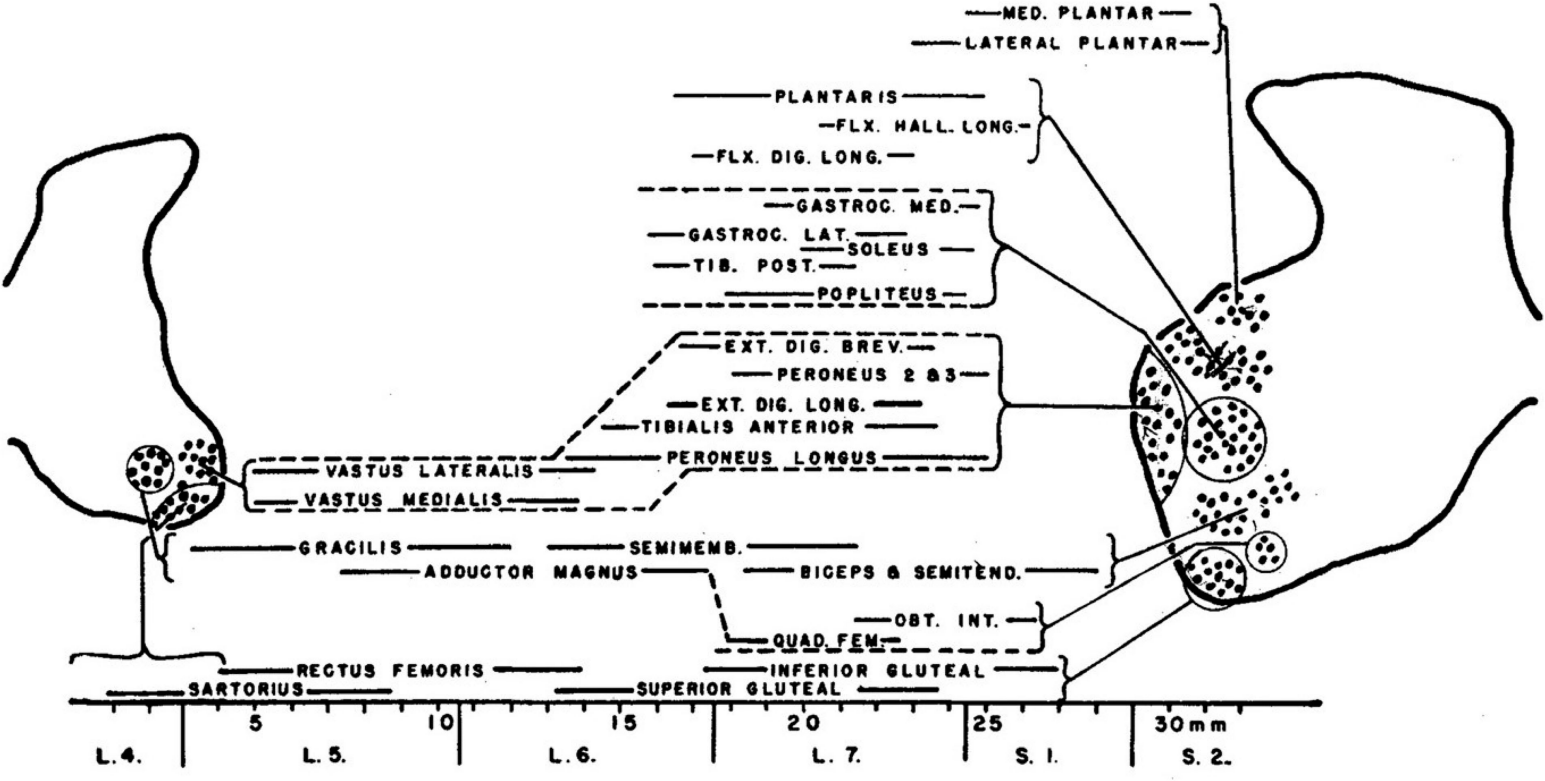
The original characterization of MN pools, documented by Romanes (1951) in the cat lumbar spinal cord.

**Figure 2:**
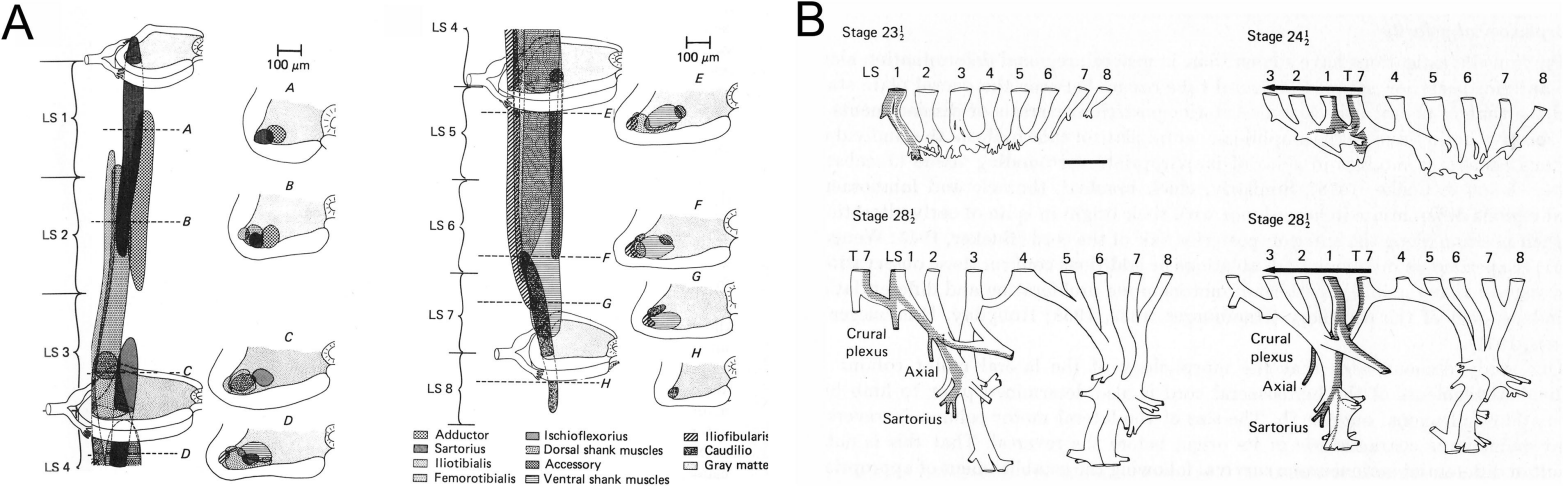
**(A)** MN pools characterized in the chicken embryo by Landmesser (1978). **(B)** Spinal cord reversal experiments showed that MN axons entering the lumbar nerve plexus at an ectopic position could correct their trajectories within the plexus, indicating that MN identity was linked to axon outgrowth (from Lance-Jones and Landmesser, 1980).

Hodological patterning begins (historically) and ends (anatomically and physiologically) with the final motor output of the spinal cord: the muscle-specific clusters of somatic motoneurons known as motoneuron pools. First described in the cat by Romanes (1951), motoneuron pools are topographically organized according to the muscle innervated (Figure 1), a feature revealed by retrograde labeling techniques (initially through retrograde degeneration following selective muscle denervation, later using retrograde axonal tracers injected into individual muscles, reviewed in Jessell et al., 2011). The hodological relationship between position in the spinal cord and axonal pathway in the periphery was established through a series of studies carried out by Lynn Landmesser and colleagues in the chicken embryo (Landmesser, 1978; reviewed in Landmesser, 1980, 2001). These showed that the axons of motoneurons (MNs) residing in a specific MN pool were able to navigate through the peripheral nerve plexus and towards the appropriate muscle, even in the face of manipulations that forced the axons to enter the periphery from a novel segmental level (Figure 2). Together with similar experiments in zebrafish (Eisen, 1991), this led to the idea that positional cues within the spinal cord during a critical window of development imbued the MNs with muscle-specific identities and equipped their axons with the capacity to respond selectively to molecular guidance cues in the periphery (Landmesser, 2001).

**Figure 3:**
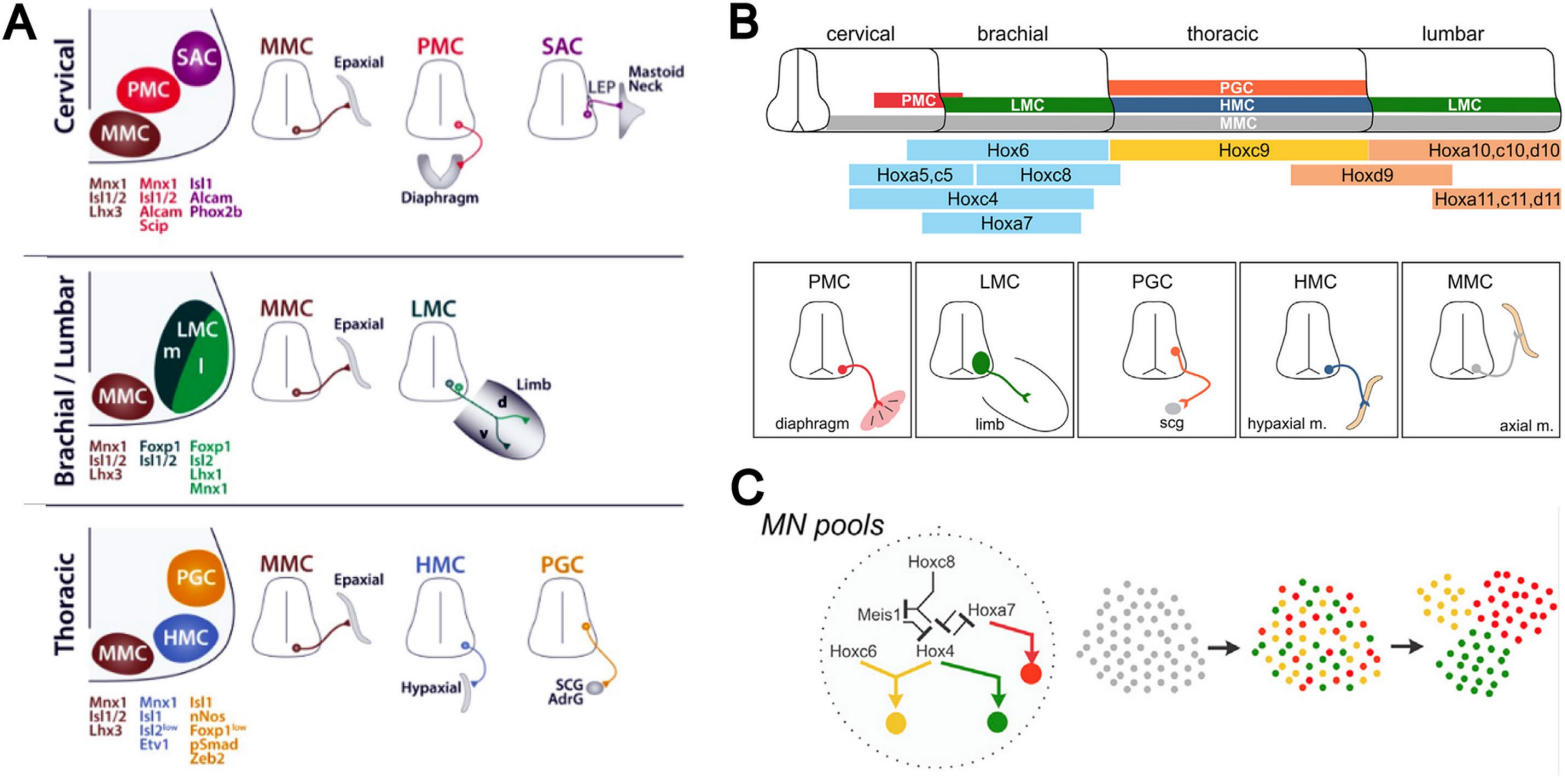
**(A)** Transcriptional signatures distinguish principal MN columns (medial motor column, MMC; lateral motor column, LMC; hypaxial motor column, HMC; phrenic motor column, PMC; spinal accessory motor column, SAC) and subpopulations (somatic versus preganglionic, PGC; medial versus lateral portions of LMC, LMC_m_ and LMC_l_). **(B)** Different MN columns relate to differential expression of Hox genes along the longitudinal axis of the spinal cord. **(C)** Cross-repressive interactions among Hox transcription factors underlie the allocation of MNs to these columns. Activation of downstream transcriptional networks, culminating in differential expression of cadherins and interactions with chemoattractive and chemorepulsive factors within the spinal cord, leads to specification of muscle-specific MNs and their aggregation into MN pools. Adapted from Philippidou and Dasen (2013).

Later studies introduced additional complexity to this idea. Assessment and manipulation of transcription factor gene expression in spinal MNs identified combinatorial transcriptional signatures associated with MN pool identities (reviewed in Dasen and Jessell, 2009; see Figure 3). It was found that for certain MN pools in the mouse, key transcription factors were only expressed after the MN axons had interacted with signaling molecules encountered in the periphery (reviewed in Jessell et al., 2011). This suggests that at least in these MNs identity is not fully determined prior to axon growth along peripheral pathways. In a mechanistic link that underscores the role of transcriptional regulation in hodological patterning, the later expressed transcription factors were shown to be upstream of cadherin expression, and differential cadherin expression was linked to the aggregation of MNs into anatomically recognizable pools (reviewed in Dasen and Jessell, 2009; Jessell et al., 2011).

As a consequence of this sequence of molecular events, MN pools can be distinguished based on patterns of gene expression, in a hierarchical system that differentiates MNs according to somatic versus autonomic function, limb versus trunk innervation, proximal versus distal limb muscle innervation, and down to individual MN pools (Figure 3). Cross-repressive interactions among Hox transcription factors set up the patterning of MN columns that differ according to general peripheral target, and initiate transcriptional networks that give rise to the column-specific molecular signatures. Further differentiation into muscle-specific MNs involves downstream expression of other transcription factors, and aggregation of the MNs into MN pools involves (at least for many MN pools) differential expression of cadherins (reviewed in Dasen and Jessell, 2009; Jessell et al., 2011). Interactions between MNs and chemoattractant and chemorepellent signaling molecules within the spinal cord also contribute (Kim et al., 2015). That the columnar organization of MNs is highly conserved among vertebrates and the clustering pattern of limb-innervating MN pools is highly conserved among limbed vertebrates supports the idea that a system of molecular interactions was established early in vertebrate evolution to specify MN identity and elaborated as limbs evolved, thus creating the MN-muscle topography first described by Romanes (Philippidou and Dasen, 2013).

## Functional significance of hodological patterning of somatic motoneurons

MN pools represent the substrate by which motor commands can be appropriately disseminated to specific sets of muscles in the temporal patterns required for context-dependent movements. Their aggregation into segregated pools provides afferent, propriospinal and descending axons with a spatial template for synaptically targeting appropriate MNs. Moreover, as originally pointed out by Romanes (1951), MN pools are further grouped according to their synergistic actions on individual joints, a “minicolumnar” organization within MN columns that Romanes coined “columels.” This feature in a sense presages experimental results in the frog that led Bizzi and colleagues to propose the existence of motor primitives, wherein stimulation at specific sites leads to stereotypic movement patterns (Giszter et al., 1993; Mussa-Ivaldi et al., 1994). Indeed, the intrinsic termination patterns of sensory afferent populations tend towards transverse positional coordinates that, overlaid on MN pool positions, automatically provide sensorimotor matching at a columellar level (Sürmeli et al., 2011). Similar assessment of other premotor axon populations should resolve whether this is a general principle governing MN inputs. MN pool-selective transsynaptic mapping is starting to make inroads on this question (see section on spinal interneurons below).

## Hodological patterning of preganglionic sympathetic neurons

Another form of hodological patterning is exhibited by preganglionic sympathetic neurons, the source of sympathetic efferent projections from the spinal cord. Preganglionic sympathetic neurons are located in thoracic and upper lumbar spinal segments and most of them send their axons into the sympathetic trunk, where they project either rostrally or caudally (the remainder project past the sympathetic trunk directly to more peripheral ganglia, as the splanchnic nerves).

**Figure 4:**
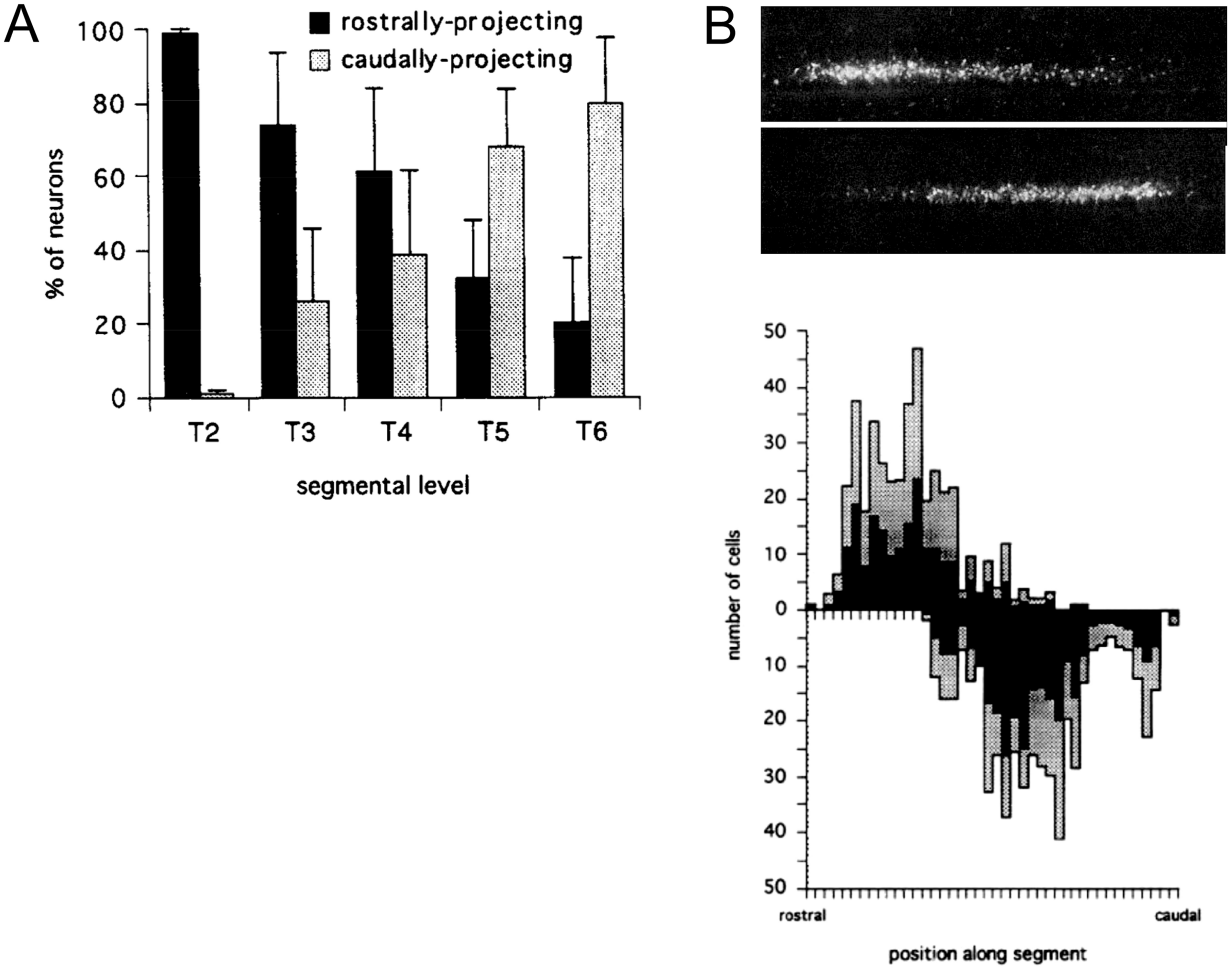
Segmental and intrasegmental bias in the projection trajectory of sympathetic preganglionic neurons, demonstrated here by retrograde tracing in the chicken embryo (Forehand et al., 1994). **(A)** Systematic shift in proportion of sympathetic preganglionic neurons projecting rostrally and caudally in the sympathetic trunk. **(B)** Upper panel shows aligned, separate images of rostrally (upper) and caudally (lower) projecting preganglionic sympathetic neurons in a mid-thoracic segment. Lower panel shows histograms of the same (rostrally projecting above x-axis, caudally projecting below), averaged for several segments (black bars show average values and grey bars show standard deviations). There are roughly equal numbers of rostrally and caudally projecting neurons in mid-thoracic segments, but they are skewed towards rostral and caudal ends of the segment, respectively.

**Figure 5:**
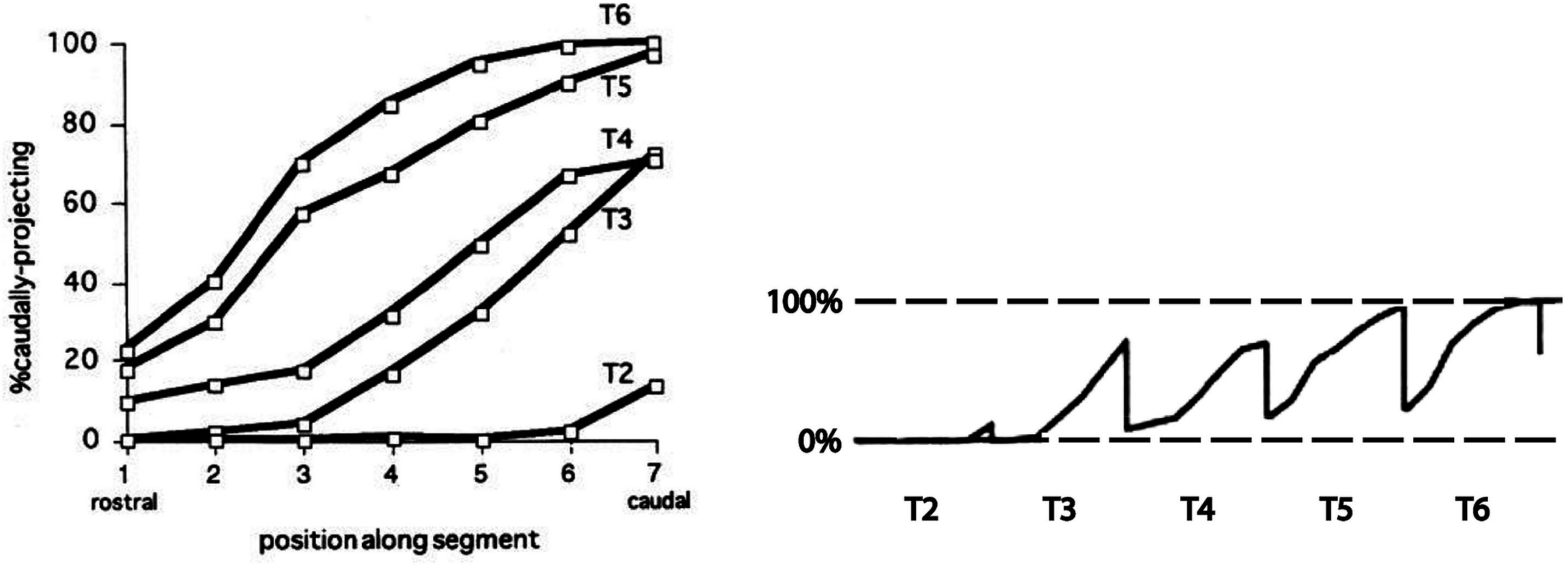
Segmental shift in intrasegmental distributions of sympathetic preganglionic neurons. Left panel shows the percentage of caudally projecting sympathetic preganglionic neurons as a function of rostrocaudal position within the segment, for the T2-T6 segments of the chicken embryo. Right panel shows these intrasegmental distributions in series, underscoring the sharp transition in projection direction at each segmental boundary. Adapted from Forehand et al. (1994).

Early physiological and anatomical studies had demonstrated a segmental shift in the proportion of preganglionic neurons that projected rostrally versus caudally in the sympathetic trunk (Njå and Purves, 1977; Lichtman et al., 1980; Rubin and Purves, 1980). The most rostral thoracic segments are dominated by preganglionic neurons that project rostrally in the trunk, the most caudal thoracic segments are dominated by preganglionic neurons that project caudally, and the relative proportions of rostrally and caudally projecting neurons shift gradually moving from rostral to caudal segments (Figure 4). To assess this relationship in more detail, Forehand et al. (1994) employed differential retrograde tracing to show that this segmental shift involved an intrasegmental bias in the location of rostrally and caudally projecting preganglionic neurons (Figures 4, 5). In each segment, rostrally projecting preganglionic neurons are skewed towards the rostral end of the segment, and caudally projecting neurons are skewed towards the caudal end of the segment.

The intrasegmental skewing of rostrally and caudally projecting preganglionic neurons leads to a sharp transition at each segmental boundary, from a large proportion of caudally projecting neurons at the caudal end of a segment to a large proportion of rostrally projecting neurons at the rostral end of the next segment (Figure 5). This was the first example of an intrinsic segmental patterning in the avian and mammalian spinal cord. How this pattern is established remains unclear. It is likely, given the role of Hox genes in patterning somatic motoneurons, that Hox genes are also involved in patterning the preganglionic sympathetic neurons, but this has not been tested directly. Support for that notion is the finding that the intrasegmental pattern is disrupted by manipulation of retinoid signaling in the thoracic cord. Retinoid signaling is a major regulator of Hox gene expression. Increasing retinoic acid levels in rostral thoracic segments decreases the proportion of rostrally projecting neurons in those segments, and decreasing retinoic acid levels in caudal thoracic segments increases the proportion of rostrally projecting neurons in those segments (Forehand et al., 1998).

## Functional significance of hodological pattering of preganglionic sympathetic neurons

Sympathetic ganglia in the sympathetic trunk innervate different peripheral targets, in a rough rostrocaudal pattern (although innervation of skin and vasculature is more divergent). The most rostral ganglia innervate targets in the head and neck region, and the most caudal ganglia innervate targets in the pelvic regions, with intermediate ganglia innervating thoracic and abdominal targets. Descending projections involved in sympathetic control can thus selectively regulate peripheral targets by modulating activity in a segmental fashion to direct traffic to postganglionic neurons in specific ganglia. The segregation of rostrally and caudally projecting preganglionic sympathetic neurons in each spinal segment translates into a segregation of sympathetic outflow directed to ganglionic targets above and below the segment. This affords descending axons the possibility to regulate and coordinate autonomic responses in different body regions by differentially distributing synaptic contacts along the rostrocaudal axis of each segment. The extent to which this is implemented remains to be tested by assessing the precise spinal termination patterns of specific descending autonomic activation pathways.

## Hodological patterning of spinal interneurons and projection neurons

The first hodological assessment of mammalian spinal projection neurons and interneurons (INs^1^) was made by Anne Lill Eide and I at the University of Oslo in a collaboration with Ole Kiehn and Ole Kjærulff of the Panum Institute in Copenhagen (Eide et al., 1999). In contrast to earlier anatomical studies, we employed strictly unilateral lipophilic dye applications to an entire hemisection in fixed preparations of the neonatal rat spinal cord to differentially label contralaterally-projecting INs and ipsilaterally-projecting INs. Through this approach we could show that contralaterally-projecting and ipsilaterally-projecting INs occupy different, characteristic domains within the transverse plane. Moreover, we could distinguish those with axons ascending versus descending in the spinal cord. Based on these findings, Anne Lill and I introduced the hodological nomenclature of aCIN/dCIN and aIIN/dIIN classes (CIN = commissural IN, IIN = ipsilaterally-projecting IN, a = ascending axon, d = descending axon), now widely used in the characterization of spinal IN types defined by other attributes such as progenitor origin.

In a follow-up study, I devised a differential labeling approach to directly compare the relative locations of aCINs and dCINs using fluorescent conjugated dextrans, which we had introduced as a tracing technique some years earlier for the express purpose of distinguishing neurons projecting along different pathways or to different target regions (Glover et al., 1986). In this study, again in collaboration with Ole Kiehn, the differential labeling approach also enabled visualization of CINs that have both ascending and descending axons, which we coined adCINs (Stokke et al., 2002).

**Figure 6:**
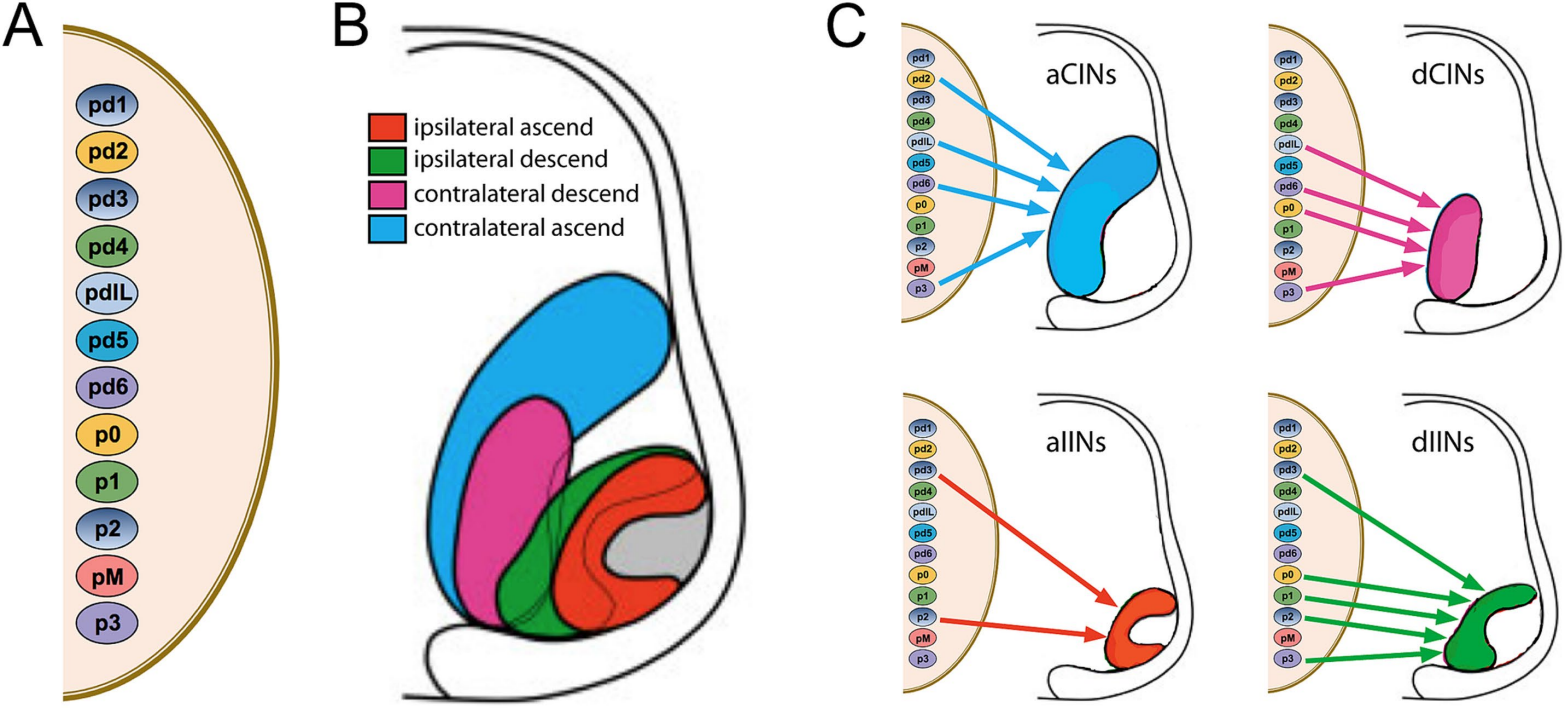
Relationship between dorsoventral progenitor origin and hodological phenotype in spinal interneurons and projection neurons. **(A)** Dorsoventral progenitor origins are indicated by the small color-coded ovals. **(B)** The nested domains of the four hodologically defined classes of interneurons and projection neurons are indicated in the hemisection of the embryonic spinal cord to the right. The outer region of the hemisection represents the white matter, and the hodological domains are located predominantly within the ventral and intermediate regions of the grey matter (the gray domain indicates the location of the somatic MNs). **(C)** Contributions from the different progenitor origins to each of the different hodological phenotypes. Note that there is a temporal relationship and phenotypic overlap between INs derived from pd4 and the dorsal part of pdIL, and between INs derived from pd5 and the ventral part of pdIL. Adapted from Nissen et al. (2005).

Relationships between hodology (axon pathway) and the molecular specification of IN identity came in yet a later study from my lab, in which we used the same differential labeling approach over a span of prenatal development (Nissen et al., 2005). In the meantime, it had become clear that spinal INs originate from several different neural progenitor subpopulations, arranged in dorsoventral sequence along the proliferative zone of the developing spinal cord (reviewed in Goulding, 2009, and extended to the human spinal cord by Rayon et al., 2021). In Nissen et al. (2005), we were able to show that aCINs, dCINs, aIINs and dIINs are systematically arrayed at an early stage of development in nested domains roughly centered on the ventral horn. This indicated a coherent relationship between axon trajectory and soma position at this early stage (the nested relationship becomes less clear with further development as the transverse area of the spinal cord increases, evidently through differential dispersal of the INs, see Eide et al., 1999). The notable conclusion is that INs that originate from multiple neural progenitor subpopulations coalesce into four hodological clusters, indicating a migratory choreography that collects INs of like axonal projection (Figure 6; note that the pMN progenitors generate MNs, not INs).

The hodological pattern has some interesting features. First, in the ventral and intermediate regions of the spinal cord, CIN domains are located medial to IIN domains at early stages, meaning that IINs are on average in closer proximity to MNs. Second, the domains of INs with ascending projections (aCINs and aIINs) sandwich the domains of INs with descending projections (dCINs and dIINs). With respect to the number of contributing progenitor subpopulations, the aIINs derive from only two progenitor subpopulations whereas each of the other hodological classes derives from at least 4 progenitor domains. Note that Figure 6 does not show the most dorsal population of aIINs, constituting a subpopulation of spinocerebellar neurons, or the most dorsal population of aCINs, constituting a subpopulation of spinothalamic neurons. These are not included in the nested domains that are shown in Figure 6 and derive, respectively, from pd1 and pd2 progenitors. Third, all 4 hodological IN classes derive from a combination of dorsal (pd) and ventral (p) progenitor subpopulations. Fourth, none of the individual progenitor subpopulations gives rise to a single hodological IN class; each gives rise to at least 2 of them (note that 2 progenitor subpopulations, pd4 and pd5, are not shown to contribute to any of the 4 hodological classes; these give rise to INs that have very short axons (respectively ipsilateral and contralateral) and thus have not been classified as ascending or descending).

What directs the migratory assembly of the hodological IN classes remains unknown. Whatever the mechanism, it clearly establishes a hodological pattern in which axon trajectory is linked to soma position, and through this to specific developmental origins based on differential gene expression in progenitor cells.

It is important to note that in these studies, the term “interneuron” has been used in a very general sense, encompassing local (intrasegmental) INs, propriospinal neurons (neurons with intersegmental axons restricted to the spinal cord), and projection neurons (with axons projecting to supraspinal levels). The reason is that the retrograde labeling used to distinguish hodological subtypes does not readily discriminate between neurons with longer versus shorter axons. Indeed, further anatomical studies suggest that there may not be sharp distinctions in the length of spinal neuron axons, but rather a gradation, such that widely used terms such as local and propriospinal, and more specific terms such as “long ascending propriospinal neurons” (LAPNs; English et al., 1985, Reed et al., 2006), are arbitrary and useful primarily in an operational sense. Nevertheless, these different classes were distinguished by Nissen et al. (2005), and the use and validity of the terms is discussed in detail there.

An important point is that the hodological pattern that is evident at early developmental stages becomes less clear with further development, due to gradual dispersal of the INs. Passive dispersal is to be expected as the volume of the neuropil increases, but active migration and aggregation of neurons may also contribute, as is known to occur for spinal MNs and other neuron types, such as reticulospinal and vestibulospinal neurons (Diaz and Glover, 2022). Thus, revealing hodological patterning will typically require observation during an appropriate window of developmental time.

## Functional significance of hodological patterning in spinal interneurons

Given the substantial functional and connectional diversity of spinal INs, it is obvious that hodological classification cannot be linked to highly specific functions within spinal neuronal circuits. So what is the functional significance of a clustering of spinal INs according to hodology? To provide some perspective, we can begin by briefly examining the functional significance of spinal IN classes as defined by progenitor origin.

The discovery of molecularly distinct progenitor subpopulations was understandably exciting, as it indicated a hitherto unappreciated subdivision of spinal INs into smaller subpopulations that might be linked to specific functions. Indeed, some progenitor-derived IN subpopulations appear to have rather circumscribed roles, such as the spinocerebellar neurons that derive from the pd1 progenitors, and the spinothalamic neurons that derive from the pd2 progenitors. Early studies that examined the role of INs derived from *ventral* progenitor subpopulations also sought to define distinct functions. To some extent this was borne out by findings that specific combinations of axon projection and neurotransmitter phenotype could be linked to progenitor origin. For example, all pd6-derived (dI6) INs are evidently inhibitory with relatively long contralateral axons (CINs), all p1-derived (V1) INs are evidently inhibitory with relatively short ipsilateral axons (IINs), and all p3-derived (V3) INs are evidently excitatory and the majority have commissural axons (CINs, although a minority are IINs; Blacklaws et al., 2015). Compelling evidence for functional attributes have come from genetic manipulations of specific IN subpopulations in mice. Thus, dI6, V0 and V3 INs, being commissural, have been linked to left–right alternation during locomotion (reviewed in Kiehn, 2016), and the V1 INs include the well-known inhibitory Renshaw cells (which mediate recurrent inhibition of MNs) and Ia inhibitory INs (which mediate inhibition of MNs to antagonistic muscles in the stretch reflex) (Sapir et al., 2004; Alvarez et al., 2005). Perhaps the most compelling functional differences that have been revealed in the context of locomotion relate to speed regulation. Here, dorsal and ventral subdivisions of the V0 CIN population, V0d and V0v, which are, respectively, inhibitory and excitatory, effect left–right alternation at low frequencies and high frequencies, respectively (reviewed in Kiehn, 2016). However, even this functional effect is difficult to parse unambiguously, since changes in speed may involve differential recruitment of specific IN classes but also differences in activity (impulse frequency and pattern) within IN classes.

The upshot of function-ascribing characterizations is that most progenitor-specific IN subtypes appear to have multiple functions. Indeed, many of the functions that have been studied are linked to locomotion in hindlimb-innervating segments and yet the same IN subclasses exist at other spinal levels where locomotion is not a central feature and where other non-limb-related functions (such as autonomic functions) are uniquely expressed. In addition, as is evident from some of the examples above, individual progenitor-specific IN subpopulations have been shown to encompass subgroups differing in neurotransmitter phenotype, axon trajectory and location in the transverse plane. In some cases, these distinctions, together with aspects of developmental timing, have led to redefinition of some of the progenitor subpopulations (for example, combining pd4 with the dorsal part of pdIL, and combining pd5 with the ventral part of pdIL). Lastly, RNA sequencing and other approaches demonstrate further parcellation into many more molecularly distinct subpopulations. For example, CINs, which *in toto* derive from 5 different progenitor domains, have been found to express additional molecular heterogenity (Tulloch et al., 2019), and GABAergic INs, which *in toto* derive from 5 to 6 different progenitor domains, comprise upwards of 50 molecularly distinct subpopulations (Gabitto et al., 2016). The V1 INs exhibit substantial heterogeneity in transcription factor expression, allowing fractionation into diverse subpopulations (Bikoff et al., 2016; Sweeney et al., 2018; Worthy et al., 2024). V3 INs can similarly be subdivided according to transcription factor expression (Deska-Gauthier et al., 2024). The pd1-derived spinocerebellar neurons comprise at least 8 molecularly distinct subpopulations (Baek et al., 2019, see below). A global single cell-RNAseq analysis of glutamatergic and GABAergic INs further underscores the transcriptional heterogeneity of each cardinal progenitor-derived IN subtype (Osseward et al., 2021).

On this backdrop, the classification of spinal INs into hodological subpopulations represents a much more general categorization than classification by progenitor origin and further parcellation according to transcription factor expression and neurogenesis. Moreover, hodological grouping has obvious functional implications, because the side and direction of axon projections necessarily contribute to determining which targets the INs influence postsynaptically. Thus, aCINs and aIINs include propriospinal neurons that link activity in caudal segments to activity in more rostral segments, as well as all projection neurons that transmit information from the spinal cord to the brain, whereas dCINs and dIINs include propriospinal neurons that link activity in more rostral segments to activity in more caudal segments. CINs link activity on one side to activity on the other. The hodological groups thus represent basic connectional components: if a network function requires activity in a hindlimb-innervating segment to influence activity in a forelimb-innervating segment on the opposite side, either an aCIN or a combination of an intrasegmental CIN and an aIIN can be envisioned to do the job. If activity in MNs within an upper lumbar segment needs to alternate with activity in MNs within a lower lumbar segment on the same side (for example in flexor-extensor alternation within a hindlimb), dIINs and/or aIINs are needed. These relationships provide an immediate entry point for determining which INs are involved in a given type of network.

There are also hints that axon length might be organized positionally within hodological IN subclasses. Kasumacic et al. (2015) used sequential retrograde labeling from different lumbar segmental levels to assess the internal organization of dCINs projecting to lumbar segment (L)5, and found that dCINs with different axon lengths have a nested distribution along the dorsoventral axis. Although limited in scope, this finding suggests that axon length may be coded positionally in a graded fashion within each hodological class.

Taking the relationship between location and connectivity to a higher level prompts the question of whether the location of INs in each hodological class is linked to the types of synaptic connections they make. The discovery of “movement primitives” in the frog spinal cord by Bizzi and colleagues, wherein stimulation at specific sites elicits stereotypical movements whose linear combination might provide the basis for more complex motor behaviors, suggested that spinal INs at specific locations might be linked to specific motor outputs (Giszter et al., 1993; Mussa-Ivaldi et al., 1994). Support for this idea came from first-generation monosynaptic retrograde viral tracing strategies in mice that purported to show a segregation of INs immediately presynaptic to, respectively, extensor and flexor limb muscles, with extensor MN-innervating INs located on average more lateral to flexor MN-innervating INs (Tripodi et al., 2011; Takeoka and Arber, 2019; reviewed in Ronzano et al., 2022) although some of the results obtained conflicted with results from another transsynaptic tracing study (Coulon et al., 2011). However, more carefully designed second-generation monosynaptic retrograde tracing convincingly demonstrates intermingling of INs presynaptic to extensor and flexor MNs, indicating that the previously reported segregation was likely artifactual (Ronzano et al., 2022). But even this does not fully resolve the question, since all of these monosynaptic retrograde tracing studies have been performed in postnatal mice, and none of them can rule out a greater degree of segregation at embryonic stages that is subsequently blurred by relative cell movements.

In one instance, the spinocerebellar neurons, transcriptional heterogeneity has been directly correlated to patterns of synaptic connectivity. Spinocerebellar neurons have long been known to be organized anatomically into groups whose rostrocaudal locations in the spinal cord are differentially linked to the muscle-specific proprioceptive sensory inputs they receive (expected, given the rostrocaudal distribution of the muscles giving rise to the proprioceptive inputs), and to termination patterns in the cerebellum (a less intuitive relationship, but likely related to cerebellar somatotopy and to differential sensorimotor processing) (reviewed in Baek et al., 2019). At least some of the 8 transcriptionally defined subpopulations of spinocerebellar neurons identified by Baek et al. (2019) map onto anatomically defined spinocerebellar groups, and disruption of underlying patterning genes (specifically *Hoxc9* and *Hoxc8*) alters the presynaptic and postsynaptic connections made (Baek et al., 2019).

As we shall see in later sections on hindbrain projection neurons, the degree of segregation exhibited by spinocerebellar neurons is not uncommon for projection neurons. Thus, it may be that hodological patterning is more distinct for neurons with long axons, since the challenge of finding appropriate synaptic partners arguably increases with distance, requiring more robust cues for axon guidance and target selection. The shorter the axon, the greater may be the tolerance for initial imprecision.

To summarize, whereas progenitor origin is a diversifying categorization for spinal INs and projection neurons, hodological class is a unifying categorization. Progenitor origin alone predicts neither connectivity nor function (these attributes must be elucidated piecemeal). By contrast, hodological classification immediately predicts connectivity in broad strokes, and through this provides definite implications about function. However, to fully elucidate hodological patterning, studies may need to be carried out at embryonic stages to avoid missing a pattern that later becomes blurred by relative movement. That the different hodological classes of spinal INs occupy different spatial domains during an early developmental period suggests at least a transient function in organizing synaptic connectivity, although the link remains obscure.

## Hodological patterning of bulbospinal neurons

**Figure 7:**
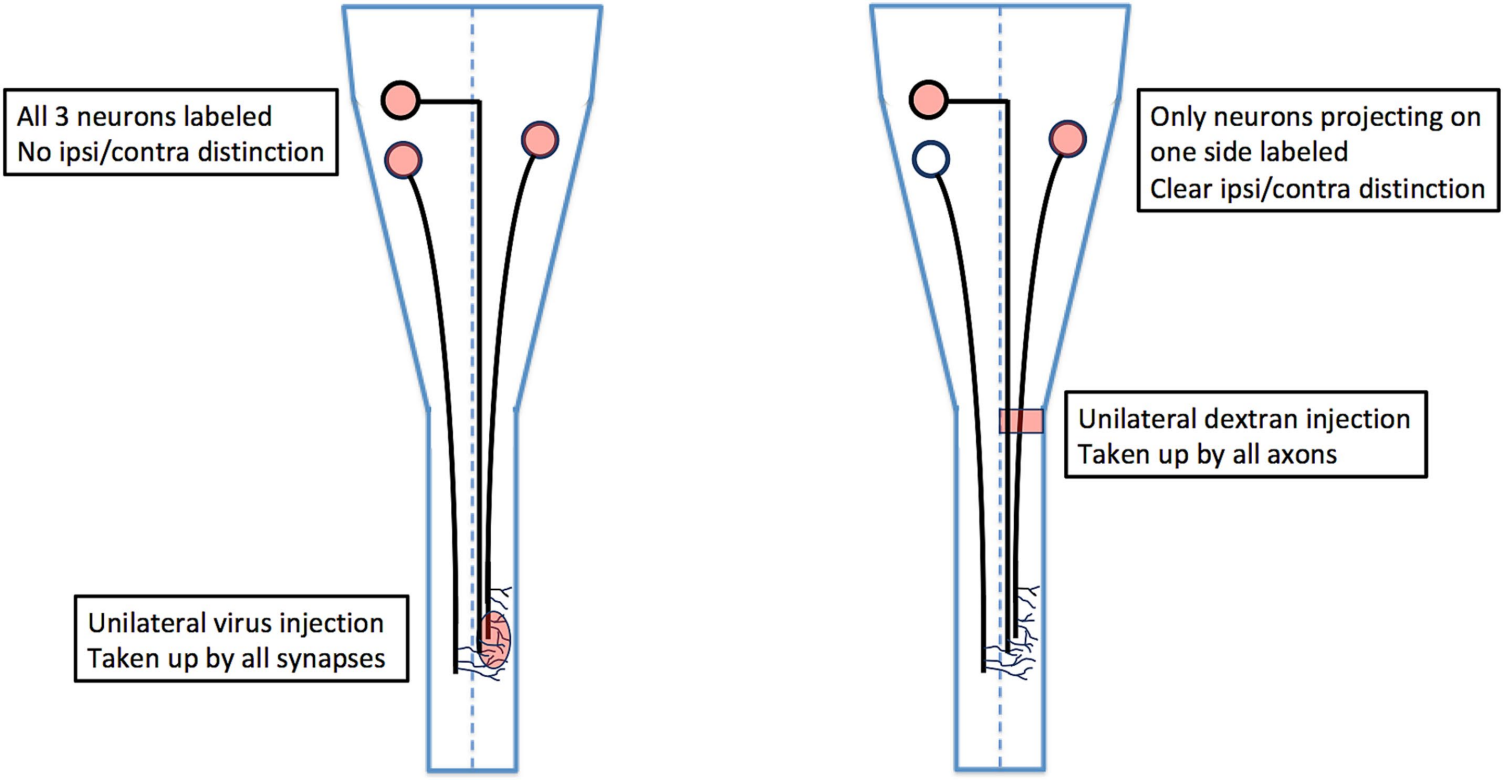
Comparison of viral tracing *in vivo* with conjugated dextran tracing ex vivo. Viral tracers are taken up by axon terminals, which means that if an axon extends collateral branches across the midline, it will be labeled from the side opposite to the side on which it projects. For this reason, a unilateral virus injection as shown will label retrogradely all three of the projection neurons, and it will not be possible to distinguish which of these projects an ipsilateral axon versus a contralateral axon (unless the axons are traced in their entirety from terminal to soma). By contrast, ex vivo labeling with conjugated dextrans restricts labeling to axons projecting on one side of the cord, thus unambiguously distinguishing ipsilaterally and contralaterally projecting neurons.

Many anatomical studies have characterized bulbospinal neurons (neurons residing in the pons and medulla and projecting to the spinal cord), but only a few have ensured that bulbospinal neurons projecting ipsilaterally versus contralaterally are unequivocally distinguished. This is because most studies have injected tracers (either conventional or viral) into the spinal cord *in vivo*, which does not prevent contamination across the midline or the combined labeling of ipsilateral and contralateral axon collaterals (see Figure 7). These confounding factors can only be eliminated by either lesioning contralateral axons in conjunction with labeling, or by using *ex vivo* preparations in which the labeling can be confirmed as exclusively unilateral. When this is done, the locations of ipsilaterally and contralaterally projecting bulbospinal neurons can be compared unequivocally (Figure 7).

Using this more definitive approach, hodological assessment of reticulospinal neurons and vestibulospinal neurons has been made in chicken embryos and mouse embryos and neonates, and in some cases combined with gene expression analyses to provide indications of potential molecular underpinnings (Glover and Petursdottir, 1991; Auclair et al., 1999; Díaz et al., 1998, 2003; Cepeda-Nieto et al., 2003, 2005; Pasqualetti et al., 2007; Sivertsen et al., 2014; Di Bonito et al., 2015; Lunde et al., 2019).

Reticulospinal neurons represent the largest and most diverse set of bulbospinal neurons (Perreault and Glover, 2013; Brownstone and Chopek, 2018). They are traditionally divided into pontine and medullary portions, but a more appropriate designation would be according to rhombomere of origin and/or settling, since the pons is not a true division of the hindbrain, but rather a mutable settling site for pontocerebellar neurons (Di Meglio et al., 2013). Nevertheless, because use of the pontine/medullary division is so pervasive, it is useful to maintain it (at least for the time being) as a bridge between traditional and modern neuroanatomy.

**Figure 8:**
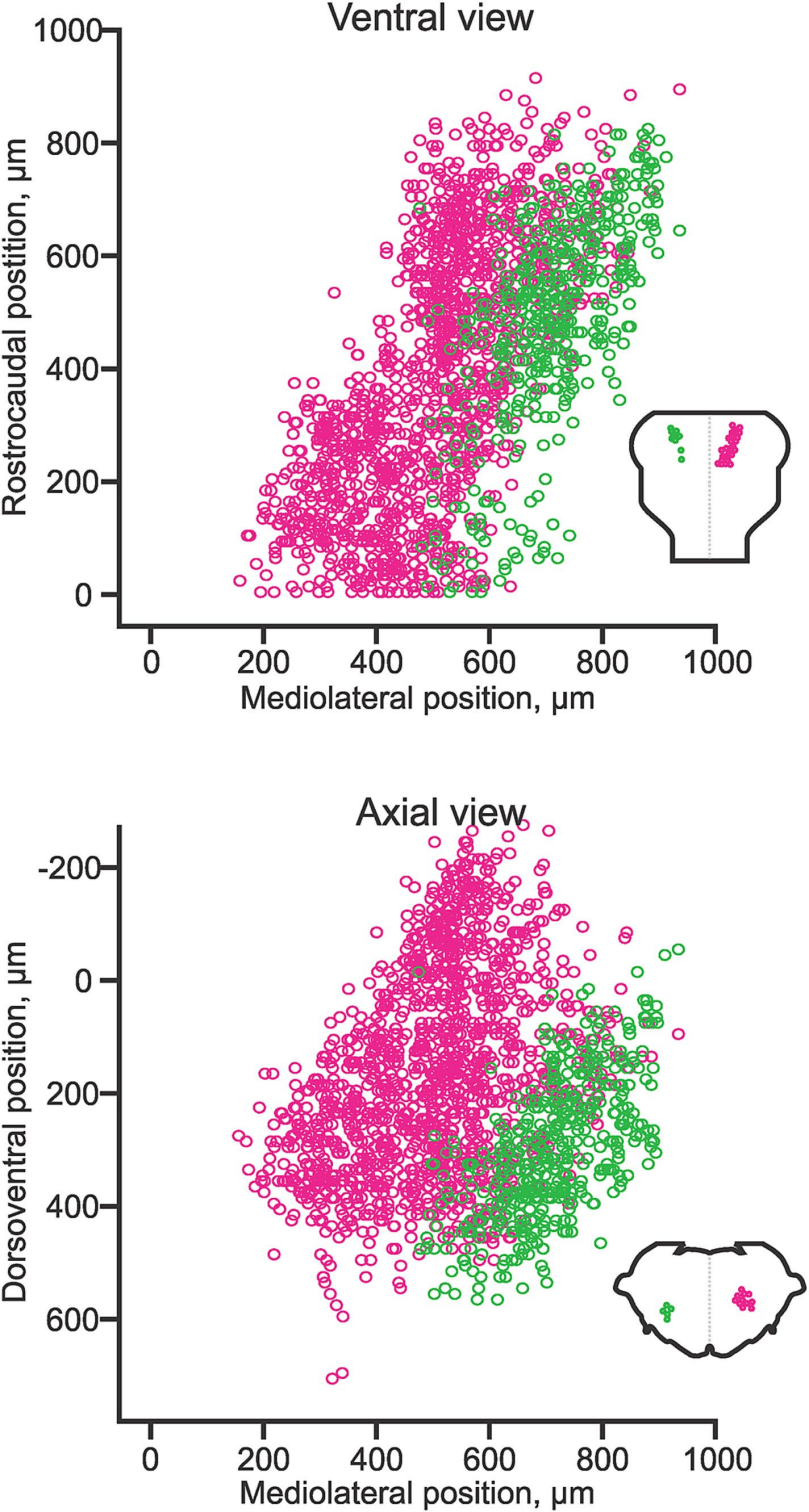
Hodological pattern of pontine reticulospinal neurons in the neonatal mouse. Ipsilaterally projecting (magenta) and contralaterally projecting (green) pRSNs occupy different and largely segregated domains. Adapted from Sivertsen et al. (2014).

The pontine reticulospinal neurons (pRSNs) in both chicken and mouse reside in (and derive predominantly from) the most rostral four rhombomeres r1-r4. In the mouse, the transition from r2 to r3 corresponds to the transition between the classically defined pRSN subdivisions nucleus pontis oralis and nucleus pontis caudalis, which corresponds to a discernible indentation in the frontal distribution of pRSNs, evidence of a difference in aggregation. In terms of hodological phenotype, the pRSNs comprise two distinct divisions, a more medial ipsilaterally projecting and a more lateral contralaterally projecting (Sivertsen et al., 2015; Figure 8). These correlate with differences in the trajectory of descending axons (more medial for ipsilaterally projecting, more lateral for contralaterally projecting; Sivertsen et al., 2015) and in the directness of connections to spinal MNs. Ipsilaterally projecting pRSNs make monosynaptic and low order polysynaptic inputs, and contralaterally projecting pRSNs make higher order polysynaptic inputs (Sivertsen et al., 2014). Thus, there is a clear correlation between soma location in the hindbrain, axon trajectory, and synaptic termination in the spinal cord. Molecular differences have yet to be assessed, but it is likely that the two projection-specific divisions arise from different dorsoventral progenitors, and thus will have different molecular signatures during their development. Moreover, given their origin from 4 different rhombomeres, there are likely to be rostrocaudal differences in gene expression as well.

**Figure 9:**
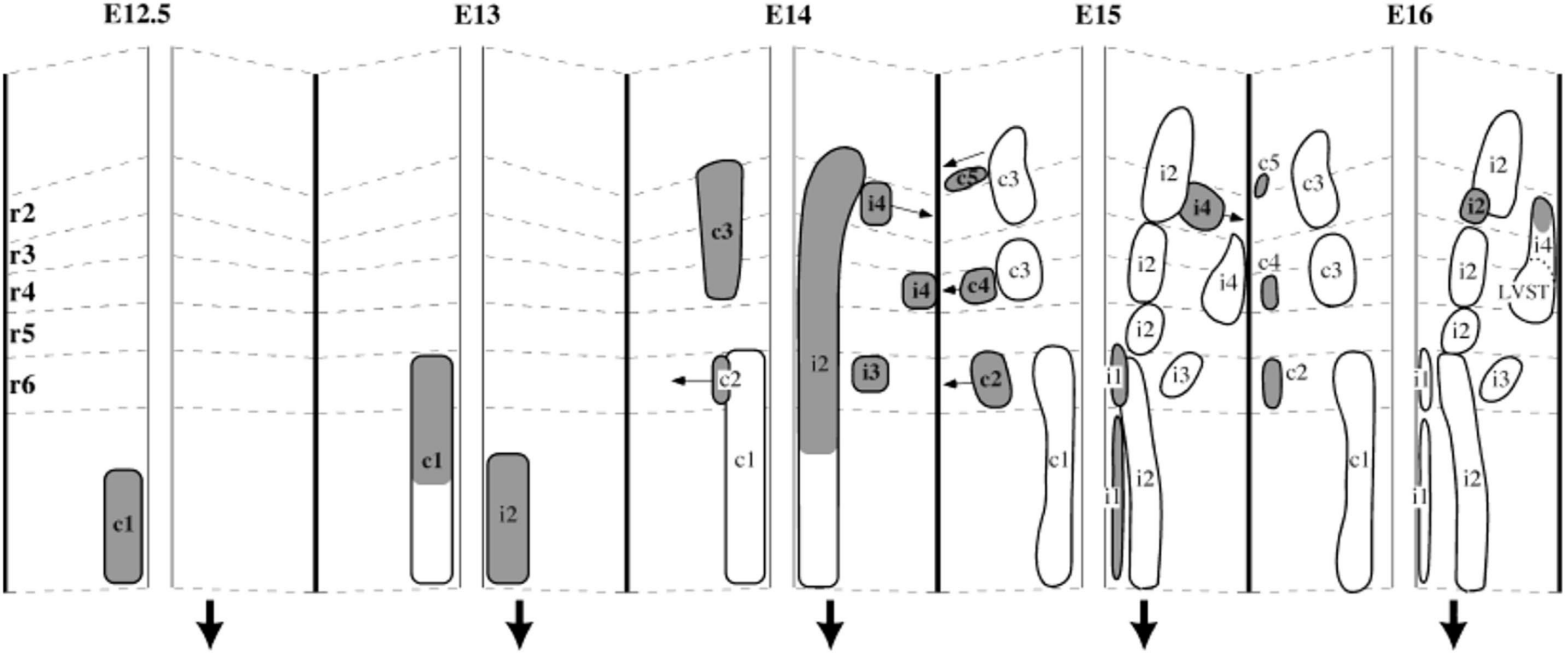
A developmental timeline showing the appearance of different reticulospinal and vestibulospinal neuron groups in the hindbrain of the rat embryo. Rhombomere (r) boundaries are indicated by dashed lines. Ipsilaterally projecting groups are shown on the right side, and contralaterally projecting groups on the left side at each timepoint (the downward-pointing arrow indicates the side on which axons descend). From Auclair et al. (1999).

The medullary reticulospinal neurons (mRSNs) comprise two main clusters located relatively medially, one projecting ipsilaterally and one contralaterally (Auclair et al., 1999; Figure 9). The ipsilaterally-projecting cluster spans from r5 to the spinal cord, whereas the contralaterally projecting cluster spans from r6 to the spinal cord in mouse, rat and chicken (Auclair et al., 1999). The lack of contralaterally projecting mRSNs in r5 appears to be a species-specific feature, as it is shifted to r3 in zebrafish and r6 in frog (reviewed in Glover, 2001). As is the case for the pRSNs, contralaterally projecting mRSNs are located more laterally than ipsilaterally projecting mRSNs. An immunohistochemical assessment has demonstrated differential expression of specific LIM homeobox proteins in ipsilaterally and contralaterally projecting mRSNs that is conserved in chicken and mouse (Cepeda-Nieto et al., 2003, 2005). A more recent single cell RNA sequencing study in the adult mouse suggests that mRSNs can be discriminated into at least 53 molecularly distinct subpopulations, partly through differential expression of LIM homeobox proteins, although this study did not distinguish between ipsilaterally and contralaterally projecting mRSNs (Winter et al., 2023). A subset of mRSNs expresses the transcription factor Chox10 and mediates a variety of effects, including initiation, modulation and cessation of locomotion, turning during locomotion, neck movements and sleep atonia (reviewed in Chopek et al., 2021).

Rhombomeric and dorsoventral origins of the mRSNs have not been assessed in detail, aside from a subpopulation of mRSNs that derive from r4 of which some migrate into r5 (Di Bonito et al., 2015). Additional fate-mapping is therefore required to bridge this major gap in knowledge. Assessment of mRSN axon trajectories indicates differential targeting of spinal funiculi, but with a more complex internal organization than is the case for the pRSNs. The hodological patterning of mRSNs is therefore less well characterized, but is likely to provide important insight into the diversity and organization of this important neuron population.

Vestibular projection neurons represent perhaps the clearest case for the link between hodology and function, as they are organized into target-specific clusters that derive from specific rhombomeres and dorsoventral progenitor domains (reviewed in Diaz and Glover, 2022). The first indications of this arose from differential retrograde labeling of vestibulospinal and vestibulo-ocular neurons (Glover, 1989; Glover and Petursdottir, 1991), and later through comparison with vestibulo-cerebellar neurons (Diaz and Puelles, 2003).

**Figure 10:**
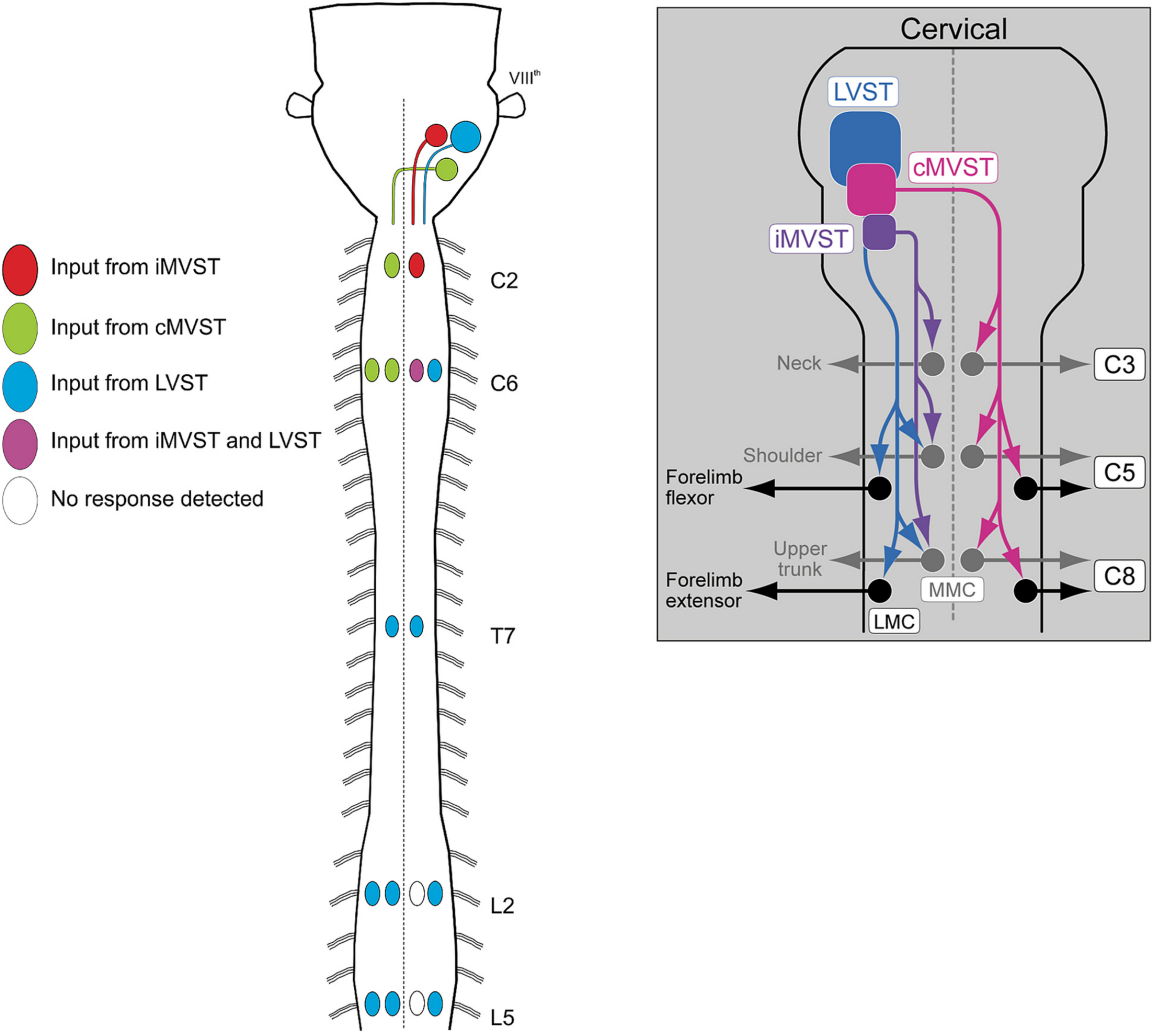
Schematic diagrams illustrating the axon trajectories and activation patterns of spinal MNs of the three best characterized vestibulospinal neuron groups, the LVST, cMVST and iMVST groups. Diagram to the left shows innervation of MNs in selected segments from cervical to lumbar levels (from Kasumacic et al., 2010). Diagram to the right provides a more detailed schematic of the MN innervation pattern in the cervical spinal cord (from Lambert et al., 2016).

The vestibulospinal projection has been the most studied. It comprises 4 different subpopulations, one that gives rise to the lateral vestibulospinal tract (LVST), two that give rise to, respectively, the ipsilateral and contralateral divisions of the medial vestibulospinal tract (iMVST and cMVST), and a fourth located in the descending vestibular nucleus that is less well characterized. The first three of these neuron groups not only differ in their axon trajectories, but also in the length of their axons and thus the spinal segments they target. The LVST neurons, which are all excitatory, extend axons along the entire length of the spinal cord, and make monosynaptic and polysynaptic connections with limb-innervating MNs in the cervical and lumbar enlargements and with thoracic MNs that control body posture, and with some neck MNs (Kasumacic et al., 2010, 2015; Lambert et al., 2016). The polysynaptic pathway includes synapses on segmentally specific sets of thoracolumbar CINs that mediate crossed connections onto contralateral MNs (Kasumacic et al., 2015). The cMVST neurons, which are a mix of excitatory and inhibitory neurons, project axons only into the cervical spinal cord where they make monosynaptic and polysynaptic connections onto neck MNs on the opposite side (Lambert et al., 2016). The iMVST neurons also have axons limited to the cervical cord, where they innervate neck MNs on the same side (Lambert et al., 2016) (Figure 10).

The LVST neurons derive exclusively from r4 (Díaz et al., 1998; Pasqualetti et al., 2007; Di Bonito et al., 2015), and from the pdB2 dorsoventral progenitors of the hindbrain (Chen et al., 2012; Lunde et al., 2019). The cMVST neurons derive from both r4 and r5, and those in r4 are in close proximity but segregated from the LVST neurons (Díaz et al., 1998; Pasqualetti et al., 2007; Di Bonito et al., 2015; Lunde et al., 2019). The r4-derived portion of the cMVST derives from one or more of the pdB1, pdB4 and pdBLa dorsoventral progenitors, whereas the r5-derived portion may derive from p0 progenitors (Lunde et al., 2019). The iMVST group derives from r6, but its dorsoventral origin has not been determined (Díaz et al., 1998; Pasqualetti et al., 2007; Di Bonito et al., 2015).

The identity of r4 and its constituent neurons depends on the expression of the homeobox gene *HoxB1*. In accordance with this, Di Bonito et al. (2015) found that knockout of *HoxB1* eliminates the LVST, the r4-derived portion of the cMVST, and several other neuron populations that derive from r4. Lunde et al. (2019) pursued the molecular specification of the LVST and cMVST neurons further, using RNA sequencing in chicken and mouse embryos to assess transcriptional profiles at early stages of differentiation. They discovered transcription factor signatures that were specific for the LVST neurons and the r4-derived cMVST neurons, not only within the vestibular neuron population, but within the entire CNS. This validates the idea that neurons that originate from a specific intersection of rhombomeric and dorsoventral domains acquire as a result a unique molecular identity, which in this case can be linked to a defined axon trajectory and pattern of synaptic connections in the spinal cord. The vestibulospinal neurons therefore exemplify hodological patterning in its purest form.

## Hodological patterning of vestibulo-ocular projection neurons

**Figure 11:**
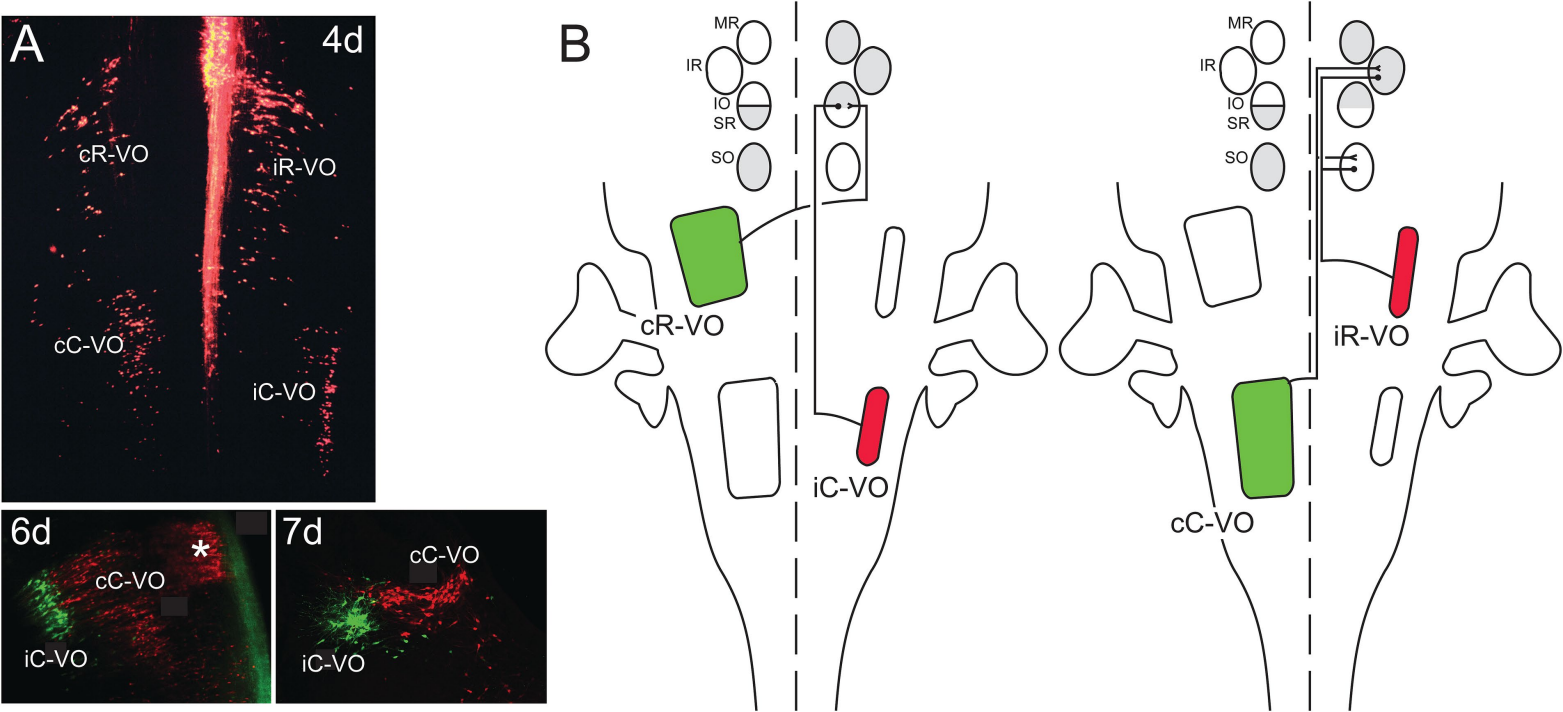
Vestibulo-ocular neurons that project to the oculomotor and trochlear nuclei are organized into coherent groups, each of which derives from a domain in the early hindbrain defined by the intersection of rhombomeres and longitudinal domains of transcription factor expression **(A)**. Shown are the 4 groups named according to their axon trajectories (i, ipsilateral; c, contralateral) and their relative locations along the rostrocaudal axis (R, rostral; C, caudal). Thus, the cR-VO group projects contralaterally and lies more rostrally. All four groups are shown at 4 days (4d) of development in a wholemount preparation of the hindbrain of the chicken embryo, a very early stage when their axons are growing towards the oculomotor and trochlear nuclei. Below, at 6d and 7d of development, the iC-VO and cC-VO groups are shown labeled differentially to highlight their segregation in the frontal plane. The groups maintain their relative positions through later development. The asterisk at 6d indicates the location of abducens interneurons, which are contiguous with the cC-VO group and project specifically to the contralateral medial rectus (MR) MN pool. **(B)** Each vestibulo-ocular neuron group projects its axons along a specific pathway to MNs innervating the extraocular muscles (MR = medial rectus, IR = inferior rectus, IO = inferior oblique, SR = superior rectus, SO = superior oblique), and terminates on a synergistic pair of MN pools, ensuring conjugate eye movements. Moreover, the vestibulo-ocular groups are organized into excitatory (green)/inhibitory (red) pairs, so that each synergistic pair of MN pools receives opposing excitatory and inhibitory inputs. Not shown are excitatory inputs to the MR (medial rectus) MNs, which originate from the abducens interneurons. Adapted from Glover (2003).

Vestibulo-ocular projection neurons mediate vestibulo-ocular reflexes by coupling activation of inner ear vestibular sensory afferents to activation of the MNs that innervate the 6 extraocular muscles. A major source of sensory input is from the semicircular canals, of which there are 3 on each side of the head, oriented in 3 orthogonal planes. By dint of the systematic arrangement of the semicircular canal planes relative to the axes of pull exerted on the eyeball by the extraocular muscles, there is a highly stereotyped link between afferent input and motor output. This link ensures that eye movements during vestibulo-ocular reflexes are equal and opposite to head movements, thus maintaining the direction of gaze. Moreover, the vestibulo-ocular reflexes operate as open loop arcs, without a requirement for sensory feedback. Thus, it was long appreciated that vestibulo-ocular projection neurons must mediate highly specific connections onto their motoneuron targets. That this involved hodological patterning was demonstrated by tracing experiments in which each coherent group of vestibulo-ocular projection neurons within the vestibular “hodological mosaic” was shown to terminate onto a specific pair of extraocular MN pools in a yoked, push-pull arrangement (Petursdottir, 1990; Jansen, 1991;^29,30^). This pattern of connectivity defines separate channels for activating or inhibiting a pair of MN pools with the result that the two eyeballs move in parallel: so-called conjugate eye movements (Figure 11).

Although this pattern has only been demonstrated for vestibulo-ocular projections to 5 of the 6 MN pools involved (those of the trochlear and oculomotor nuclei; projections to the abducens nucleus have not yet been assessed within the hodological mosaic), it clearly illustrates a link between projection neuron location, axon trajectory and synaptic connectivity. Molecular profiling of the vestibulo-ocular projection neurons has not yet been performed, but judging from the results of profiling vestibulospinal neurons (Lunde et al., 2019), it is likely that similarly specific transcriptional signatures will be revealed for vestibulo-ocular projection neurons.

## Hodological patterning of corticospinal projections

**Figure 12:**
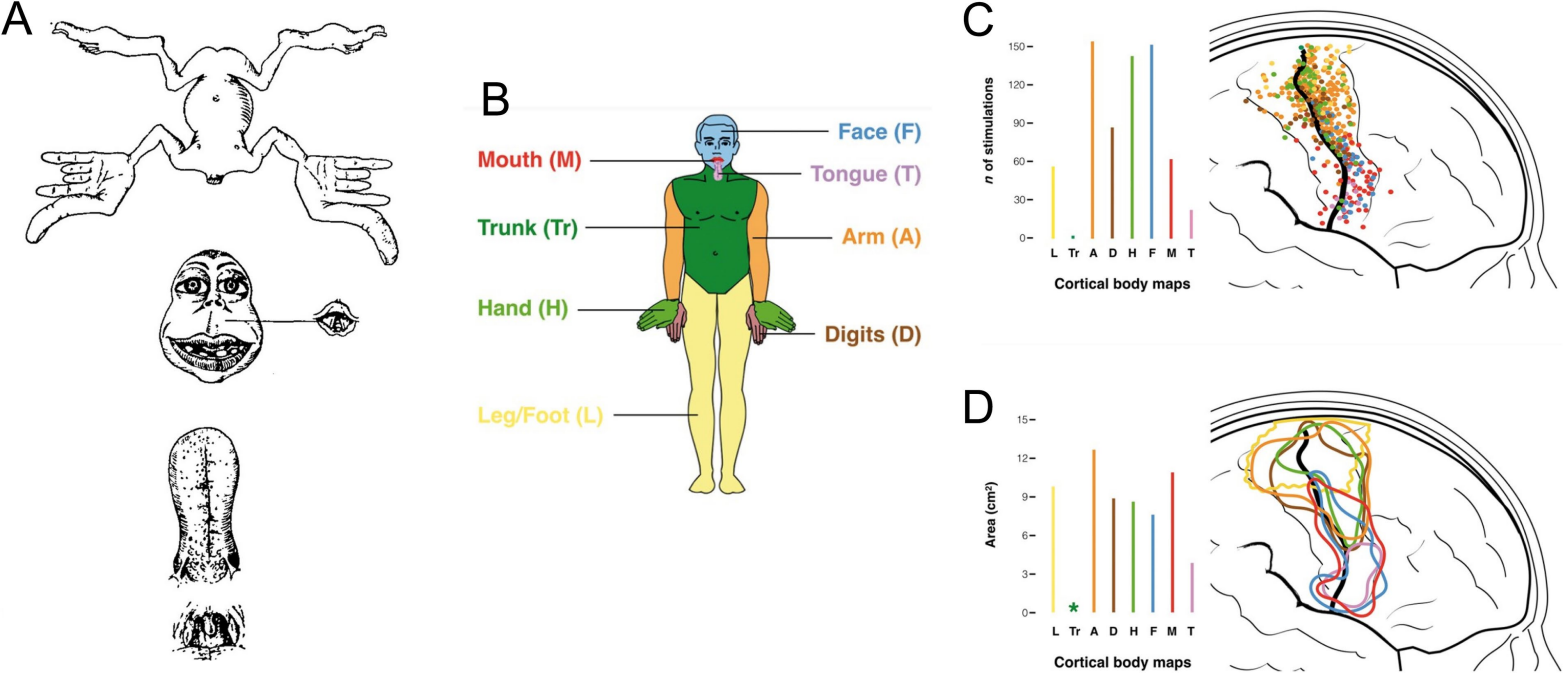
**(A)** The original motor homunculus as depicted by Penfield and Boldrey (1937). **(B)** Color coding of body regions used for somatotopic mapping of M1 shown in **(C)** (body region-specific stimulation point map) and **(D)** (circumscribed regions encompassing body region-specific stimulation points). Adapted from Catani (2017).

As for MNs, the final common output of the motor system, the motor cortex, the highest level of executive motor control, was a stage for early investigations of topographic relationships between neurons and synaptic targets. The pioneering work of Penfield and collaborators demonstrated the systematic topographic relationship between electrical stimulation points in the precentral gyrus and the sites of elicited body movement that led to the renowned concept of the motor homunculus (Penfield and Boldrey, 1937; reviewed in Catani, 2017; Figure 12). Although highly popularized and nearly indelible in the collective consciousness regarding brain organization, the motor homunculus has been subject to substantial later revision. Transsynaptic tracing studies in higher primates have demonstrated a subdivision of primary motor cortex (M1) into a separate region (“new” M1) populated by corticospinal neurons that monosynaptically innervate spinal MNs to upper limb muscles, especially hand and finger muscles (CS_MN_ neurons), and the main region of M1 (“old” M1, common to all mammals) containing corticospinal neurons that regulate spinal MN activity through weaker monosynaptic connections and indirectly through spinal INs (CS neurons; Rathelot and Strick, 2009; Witham et al., 2016). There is a high degree of overlap in the locations of muscle-specific CS_MN_ neurons (Rathelot and Strick, 2006; Figure 12). This has been surmised to be related to the coordination of motor synergies across multiple joints (Rathelot and Strick, 2009). In addition, CS_MN_ neurons exhibit heterogeneous functional properties and fire in varied relationships to spinal MN firing patterns and movement parameters, a complexity that challenges understanding of cortical movement control (reviewed in Lemon, 2019, 2024). Based on the anatomical and neurophysiological evidence, it has been postulated that the principal role of CS_MN_ neurons is to regulate fine, low force movements of the hands and digits, a requirement for the dexterity exhibited by higher primates including humans (reviewed in Lemon, 2019, 2024).

**Figure 13:**
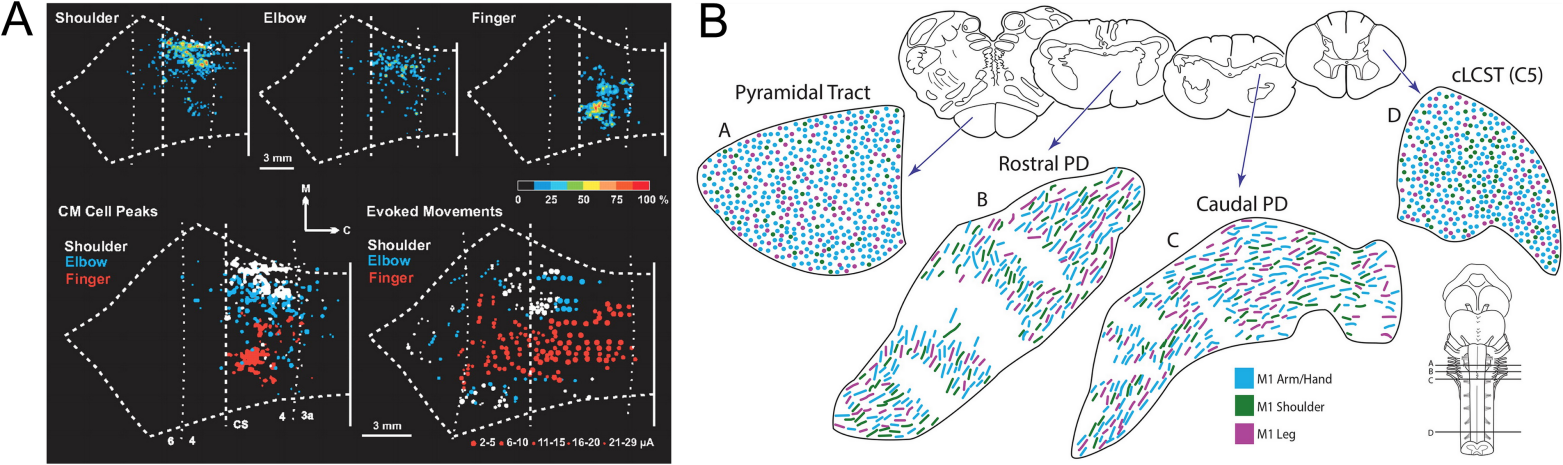
**(A)** Somatotopic overlap of stimulation points in M1 (new) From Rathelot and Strick (2009). **(B)** Somatotopic intermingling of CS_MN_ axons, as they descend through the brain stem and into the spinal cord. From Lemon and Morecraft (2023).

A general somatotopic relationship of descending corticospinal axons is maintained in higher primates as the axons descend through the internal capsule, but by the time the cerebral peduncle is reached, axons destined for MNs innervating different parts of the extremities become intermingled (Figure 13; Lemon and Morecraft, 2023; reviewed in Lemon, 2024). A slavish somatotopy is clearly not required as the CS_MN_ axons approach their spinal targets, given the complex muscle synergies that appear to be the substrate for their actions (Lemon, 2024). In addition, CS_MN_ and CS projections exhibit varying degrees of laterality in their spinal terminations depending on which limb and which joint(s) along the limb’s proximodistal axis is targeted (reviewed in Lemon, 2024). In the mouse, CS projections from caudal versus rostral regions of the forelimb area terminate differentially in the spinal cord, evidently keying in on premotor interneurons involved in reaching versus grasping, respectively (Wang et al., 2017).

The CS_MN_ and CS projections thus exhibit hodological patterning in the sense that they are organized in a coarse but systematic somatotopy and with limb-specific laterality and task-specific spinal terminations. How this patterning is established remains unclear. M1 is set apart from other cortical areas through a molecular patterning process called arealization (reviewed in O'Leary et al., 2007). Arealization and the development of subsequent somatotopic organization has been studied extensively in the somatosensory cortex (reviewed in Simi and Studer, 2018), but not in M1. One longstanding idea is that incoming thalamocortical and cortico-cortical afferents, which are already somatotopically organized, impose that organization on M1 through the same kinds of nearest neighbor axon-axon interactions and activity dependent plasticity invoked to explain somatotopy in the somatosensory cortex (Erzurumlu and Gaspar, 2012; Renier et al., 2017). If this is the case, it remains to be explained how the point-to-point somatotopy of the somatosensory projection is converted to the more highly overlapping somatotopy of M1, and the establishment of the separate representation of the upper extremity in “new” M1.

## Summary

Motor control involves the coordinated activation of different muscles in complex temporal patterns. The neural circuitry mediating this impressive choreography remains obscure despite significant advances in the anatomical, physiological, and particularly the molecular characterization of the neurons involved, from cortex to brain stem to spinal cord. A major challenge is linking molecular diversity to connectivity itself, through the specification of axon trajectories and synaptic contacts. This is the core principle of hodological patterning: bridging from gene expression, as laid out in spatial domains of neural progenitors within the developing brain and spinal cord, to the physical attributes that enable neurons to find and engage with their appropriate synaptic partners. In essence, hodological patterning can be conceived as a spatial blueprint for establishing the synaptic connections that make motor control possible.

This review has examined hodological patterning at different levels of the motor hierarchy, from cortex to brain stem to spinal cord (Table 1). These give rise to either direct (monosynaptic) or indirect (polysynaptic) commands to the final motor output – the motoneurons that innervate muscle and autonomic ganglia. We have seen that neurons at each of these levels exhibit hodological patterning. In some cases, in particular among vestibular projection neurons, the link between hodological patterning and synaptic connectivity and function within motor circuits is readily apparent. In many other cases there remain significant gaps in our knowledge about the relationship between hodological patterning and circuit function. Moving forward, a more complete account of hodological patterning should facilitate a deeper understanding of how motor circuits are constructed during development, and by extension how they operate.

**Table 1:** Types of hodological patterning exhibited by different neuron categories, and current knowledge about underlying patterning mechanisms.

	Hodological pattern	Underlying mechanisms
Somatic MNs	Segregated clusters (MN pools)	Transcriptional networks
Preganglionic sympathetic neurons	Opposing gradients (intrasegmental bias)	Not known, retinoid signaling dependent
Spinal INs/projection neurons	Nested domains	Not known
Spinocerebellar neurons	Coherent clusters at specific locations	Transcriptional networks
Reticulospinal neurons	Segregated domains of ipsi- and contralaterally projecting	Not known
Vestibular projection neurons	Rhombomeric/dorsoventral domains	Likely transcriptional networks
Corticobulbar/corticospinal neurons	Fractionated, overlapping somatotopic	Arealization followed by somatotopically patterned afferents (?)

## Footnotes

^1^For simplicity, the abbreviation IN is used hereafter both for spinal interneurons and spinal projection neurons.
